# Pre-Transplant Depression Is Associated with Length of Hospitalization, Discharge Disposition, and Survival after Liver Transplantation

**DOI:** 10.1371/journal.pone.0165517

**Published:** 2016-11-07

**Authors:** Shari S. Rogal, Gautham Mankaney, Viyan Udawatta, Matthew Chinman, Chester B. Good, Susan Zickmund, Klaus Bielefeldt, Alexis Chidi, Naudia Jonassaint, Alison Jazwinski, Obaid Shaikh, Christopher Hughes, Paulo Fontes, Abhinav Humar, Andrea DiMartini

**Affiliations:** 1 Center for Health Equity Research and Promotion, VA Pittsburgh Healthcare System, Pittsburgh, PA, United States of America; 2 Department of Surgery, University of Pittsburgh, Pittsburgh, PA, United States of America; 3 Division of Gastroenterology, Hepatology, and Nutrition, University of Pittsburgh, Pittsburgh, PA, United States of America; 4 Division of General Internal Medicine, University of Pittsburgh, Pittsburgh, PA, United States of America; 5 Rand Corporation, Pittsburgh, PA, United States of America; 6 Department of Psychiatry, University of Pittsburgh, Pittsburgh, PA, United States of America; University of Toledo, UNITED STATES

## Abstract

Depression after liver transplantation has been associated with decreased survival, but the effects of pre-transplant depression on early and late post-transplant outcomes remain incompletely evaluated. We assessed all patients who had undergone single-organ liver transplantation at a single center over the prior 10 years. A diagnosis of pre-transplant depression, covariates, and the outcomes of interest were extracted from the electronic medical record. Potential covariates included demographics, etiology and severity of liver disease, comorbidities, donor age, graft type, immunosuppression, and ischemic times. In multivariable models adjusting for these factors, we evaluated the effect of pre-transplant depression on transplant length of stay (LOS), discharge disposition (home vs. facility) and long-term survival. Among 1115 transplant recipients with a median follow-up time of 5 years, the average age was 56±11 and MELD was 12±9. Nineteen percent of the study population had a history of pre-transplant depression. Pre-transplant depression was associated with longer LOS (median = 19 vs. 14 days, IRR = 1.25, CI = 1.13,1.39), discharge to a facility (36% vs. 25%, OR 1.70,CI = 1.18,2.45), and decreased survival (HR = 1.54,CI = 1.14,2.08) in this cohort, accounting for other potential confounders. In conclusion, pre-transplant depression was significantly associated with longer transplant length of stay, discharge to a facility, and mortality in this cohort.

## Introduction

With advances in immunosuppression, standardization of surgical technique, and improved recipient selection, liver transplantation is the standard of care for advanced cirrhosis. However, long-term survival after liver transplant remains suboptimal, with only 60% of recipients surviving to 10 years [1]. Thus the identification of modifiable risk factors associated with poorer survival has been a key goal of liver transplant outcomes research. Depression is potentially one of these modifiable risk factors and is common among patient with cirrhosis with rates over twice population norms (5). Depression has been convincingly linked with poorer survival in other medical disorders (e.g. heart failure, acute myocardial infarction and stroke)[2–4]. Depression detracts from quality of life [5] and is associated with disability among patients with chronic liver disease [6], but remains undertreated both in the pre- and post- transplant settings [7, 8]. Therefore, it is critical to understand the associations between depression and outcomes after liver transplantation.

There has been limited investigation into the relationship between depression and outcomes among patients undergoing liver transplantation. Prior studies suggest that depression negatively impacts some transplant outcomes. Pre-transplant depression has been associated with decreased quality of life and decreased self-reported recovery after liver transplant [7, 9]. We found *post*-transplant trajectories of depressive symptoms in the year following transplant were associated with increased long-term all-cause mortality, particularly among recipients on inadequate or no antidepressant medications in the first post-transplant year [10]. However, using post-transplant depression as a predictor of outcomes does not allow examination of the critical events occurring in the immediate perioperative period such as length of hospital stay or disposition. Few studies of depression in the setting of transplant have assessed associations beyond survival and quality of life. We found, in a small cohort, that recipients with any history of pre-transplant depression, who were untreated at the time of transplant, had increased acute rejection after transplantation [11]. Larger studies supporting the relationship between pre-transplant depression and post-transplant outcomes, particularly early after transplant, are still lacking. Identifying potential pre-transplant predictors of poor outcomes after transplantation may enable clinicians to target individuals at risk with appropriate interventions and improve transplant outcomes. Thus, we conducted a large, single-institution retrospective analysis to understand 1) the role of pre-transplant depression in immediate post-transplant outcomes and long-term survival and 2) how antidepressant medication use at the time of transplant impact these outcomes. In particular, evaluating pre-transplant depression allowed us to examine not only long-term mortality but also proximal events such as length of stay and discharge disposition.

## Materials and Methods

This study was approved by the University of Pittsburgh IRB PRO13070515 with a waiver of informed consent due to the retrospective nature of the study. We reviewed the electronic medical records of all patients who underwent first-time liver transplantation at the University of Pittsburgh from 2004–2014 (n = 1255). Recipients were excluded if they had undergone combined organ transplant (n = 103) or had fulminant hepatic failure (n = 37). Data extracted included demographics, etiology of liver disease, hepatocellular carcinoma (HCC) status at initial transplantation, donor age (living or deceased), donor type (donation after brain death, donation after cardiac death, or living donor), cold and warm ischemia times, type of immunosuppression on transplant discharge, and model for end-stage liver disease (MELD) at transplantation. HCC was confirmed by explant pathology or history of documented HCC treatment. The etiology of liver disease was divided into hepatitis C (HCV) with or without alcohol, alcohol alone, non-alcoholic steatohepatitis (NASH), autoimmune, and other. Recipients with cryptogenic cirrhosis in the chart who were likely to have NASH based on physician notes were classified as NASH. Based on medical diagnosis codes, we collected information regarding pre-transplant co-morbid illnesses and calculated each individuals Charlson Comorbidity Index, a commonly used index of medical comorbidity developed based on one-year mortality data [12]. Outcomes collected included length of transplant admission, location of discharge (home vs. acute rehabilitation vs. long-term nursing facility), and date and cause of death.

### Depression Measures

ICD-9 diagnosis codes for all categories of depressive disorders were collected from the problem list in the electronic medical record. For the purposes of the study, if at any point prior to transplant a patient had any type of depression diagnosis present in their medical record, they were categorized as having a history of depression. Otherwise, the patient was categorized as having no history of depression.

### Statistical Analysis

R version 2.14.2 was used for all analyses. Univariable analyses were completed using Student’s t-tests for normal continuous, Wilcoxon rank-sum and Kruskal-Wallis test for non-parametric continuous, and chi-square and Fisher’s exact tests for categorical variables in order to evaluate how depression and the covariates associated with the outcomes of interest. Univariable regression models were made to evaluate the association between covariates and outcomes and then full regression models were created and then reduced using automated AIC optimization. Logistic and negative binomial regressions were used to assess factors associated with discharge disposition after transplant and transplant length of stay (LOS), respectively. Transplant survival was evaluated using Cox proportional-hazards models. All models were checked for multicollinearity using a pre-specified variance inflation factor of 5. Causes of death were evaluated by depression status and compared using Fisher’s exact tests. Non-significant covariates that were omitted from the models are noted in the table footnotes. All confidence intervals (CI) are 95% Cis.

## Results

### Patient Population

The final cohort included 1115 recipients. Table 1 shows the basic demographic characteristics of the total population and by pre-transplant depression status. The recipients were predominantly male and Caucasian with an average age of 56 and MELD score of 21. Immunosuppression was predominantly with tacrolimus (79%), followed by cyclosporine (9%), with 12 recipients on sirolimus/everolimus and 11 on other regimens on discharge from the initial hospitalization. The median follow-up time was 4.6 years (IQR = 1.8,7.6). Prior to transplant 207 (19%) had a history of depression, and those with a history of depression were significantly younger, were more likely to be female and Caucasian, and had a younger mean donor age than those recipients who did not have a history of depression.

**Table 1:** Patient Characteristics for the Cohort and by Depression Status.

	Total	No Pre-transplant Depression	Pre-transplant Depression	P
	(N = 1115)	(N = 866)	(N = 207)	
***Demographics & Comorbidities***				
**Age at transplant (mean)**	56±11	57±10	54±10	<0.01
**Female sex (n)**	404 (36%)	286 (33%)	98 (47%)	<0.01
**Race (n)**				0.04
** White**	1037 (93%)	797 (92%)	201 (97%)	
** Black**	46 (4%)	41 (5%)	5 (2%)	
** Other**	26 (2%)	24 (3%)	1 (<1%)	
**Comorbidity score (mean)**	5.3±1.4	5.3±1.4	5.3±1.4	0.94
***Liver Disease Factors***				
**MELD (mean)**	21±9	21±9	22±10	0.35
**Hepatocellular carcinoma (n)**	215 (19%)	179 (21%)	33 (16%)	0.15
**Etiology of liver disease (n)**				0.24
** Hepatitis C**	391 (35%)	310 (36%)	72 (35%)	
** Alcohol**	212 (19%)	162 (19%)	46 (22%)	
** NASH**	160 (14%)	116 (13%)	34 (16%)	
** AIH/PBC/PSC**	177 (16%)	149 (17%)	24 (12%)	
** Other**	171 (15%)	128 (15%)	31 (15%)	
***Transplant Factors***				
**Donor age (mean)**	47±18	47±18	44±18	0.05
**DCD donor (n)**	114 (10%)	88 (10%)	22 (11%)	0.94
**Living donor (n)**	187 (17%)	148 (17%)	33 (16%)	0.77
**WIT (median min)**	30 (25,36)	30 (25,34)	30(25,32)	0.55
**CIT (median hours)**	8.6(6.2,11.4)	8.5(6.2,11.4)	8.6(5.9,11.2)	0.87

Numbers may not sum to 100% due to missing data. Pre-transplant depression data could not be found for 42 patients (4% of the cohort). %s are column %s. Abbreviations: NASH = non-alcoholic steatohepatitis, AIH = autoimmune hepaitits, PBC = primary biliary cirrhosis, PSC = primary sclerosing cholangitis DCD = donation after cardiac death, wit = warm ischemia time, cit = cold ischemia time, fk = tacrolimus, is = immunosuppression

### Length of Stay

The median length of transplant hospitalization (LOS) was 14 days (IQR = 10,27). Pre-transplant depression was significantly associated with LOS in univariate and multivariate models. Pre-transplant depression was associated with LOS in univariable and multivariable models (median = 19 vs. 14 days, IRR = 1.25, CI = 1.13,1.39). Other factors associated with LOS in univariable and multivariable models included MELD score at transplant (IRR/point = 1.02, CI = 1.02,1.03). HCC was associated with decreased length of stay (median 12 vs. 15 days, IRR = 0.77 CI = 0.69,0.86). A longer LOS was associated with non-tacrolimus-based immunosuppression (median 33 vs. 13 days, IRR = 1.98, CI = 1.79,2.28). Female gender and Charlson Comorbidity Index were significantly associated with LOS in univariate but not multivariate modeling.

### Post-Transplant Discharge Disposition

In terms of initial disposition, 58 (5%) patients did not survive their initial admission and 70 (6%) survived but did not have their discharge disposition documented in the electronic medical record. Among the 987 recipients with a known discharge disposition who survived the initial hospitalization, 692 (70%) were discharged home, 234 (24%) required transfer to a long-term acute care, and 61 (6%) required skilled nursing facility (SNF). Mean pre-transplant comorbidity index was not associated with disposition after transplant to home vs. facility (5.4 ±1.4 vs. 5.3±1.4, p = 0.64). Pre-transplant depression was significantly associated with discharge to a location other than home (either acute care or long-term care facilities). Thirty-six percent of patients with pre-transplant depression were discharged to a facility compared with 25% without pre-transplant. The statistically significant predictors of long-term care requirements (discharge to somewhere other than home) in a multivariate logistic regression model included age at transplant, female gender, depression pre-transplant, and higher MELD score at transplant (Table 2).

**Table 2:** Disposition After Transplant: Primary Data and Logistic Regression Models*

	Primary Data			Univariate Analysis		Multivariate Analysis	
**Covariate**	Home	Acute Care	Long-Term Care	OR	95% CI	OR	95% CI
	(N = 692)	(N = 234)	(N = 61)				
***Demographics and Comorbidities***							
**Age**	55±10	59±10	57±8	**1.04**	**1.03,1.06**	**1.06**	**1.04,1.07**
**Female sex**	223 (38%)	99 (42%)	26 (43%)	**1.57**	**1.18,2.09**	**1.50**	**1.09,2.06**
**Depression**	119 (17%)	55 (23%)	19 (31%)	**1.66**	**1.19,2.31**	**1.68**	**1.16,2.45**
***Liver Disease Factors***							
**MELD**	20±8	24±10	28±9	**1.07**	**1.05,1.08**	**1.07**	**1.05,1.09**
**Etiology of liver disease**							
** Hepatitis C**	261 (38%)	68 (29%)	28 (46%)	—	—		
** Alcohol**	126 (18%)	50 (22%)	17 (28%)	1.44	0.99,2.11		
** NASH**	95 (14%)	35 (15%)	6 (10%)	1.16	0.74,1.78		
** AIH/PBC/PSC**	115 (17%)	37 (16%)	7 (11%)	1.02	0.67,1.56		
** Other**	95 (14%)	44 (19%)	2 (3%)	1.30	0.84,1.99		
**Hepatocellular carcinoma**	160 (23%)	29 (12%)	13 (21%)	0.56	0.38,0.81	**0.43**	**0.28,0.64**
***Transplant Factors***							
**Living donor**	136 (20%)	28 (12%)	3 (5%)	0.48	0.31,0.72		
**Non-tacrolimus immunosuppression**	52 (8%)	52 (22%)	19 (31%)	3.98	2.69,5.90	**3.77**	**2.46,5.80**

* The logistic regression model compared discharge to a location other than home vs. discharge home after transplantation

Nonsignificant variables that were not included in the table were race, comorbidity index, donation after cardiac death, donor age, ischemia times

Abbreviations: NASH = non-alcoholic steatohepatitis, AIH = autoimmune hepaitits, PBC = primary biliary cirrhosis, PSC = primary sclerosing cholangitis

### Survival

**Fig 1:**
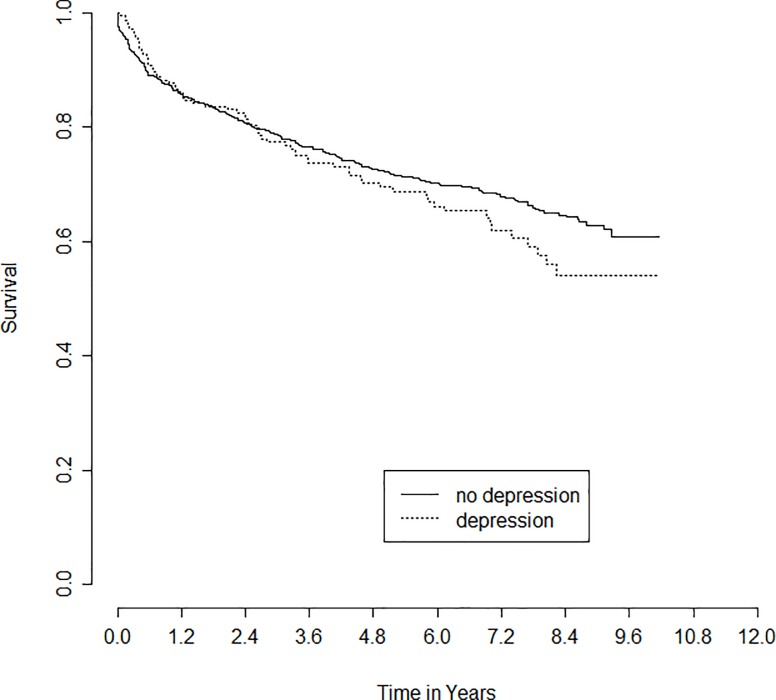
Survival by Pre-Transplant Depression Status.

Over the length of study follow-up 356 recipients died (32%). Fig 1 shows the Kaplan-Meier curves for survival of those recipients with vs. without pre-transplant depression. Depression was significantly associated with decreased survival time. In multivariable modeling (Table 3) depression was significantly associated with increased mortality. Factors significantly associated with improved survival time were younger age, other (non-White, non-Black) race, HCC, non-HCV etiology, tacrolimus-based immunosuppression, shorter warm ischemia time, lower MELD, and living donor transplant. The causes of death are shown in Table 4 both by depression vs. no depression. The only cause of death that was significantly increased among those with a history of pre-transplant depression was withdrawal of care.

**Table 3:** Post-Transplant Survival: Primary Data and Cox Proportional-Hazards Models.

	Primary Data		Univariate Analysis		Multivariate Analysis	
**Covariate**	Alive	Not Alive	HR	95% CI	HR	95% CI
	(N = 759)	(N = 356)				
***Demographics and Comorbidities***						
**Age (mean)**	54±11	59±11	**1.03**	**1.02,1.05**	**1.03**	**1.01,1.04**
**Race (n)**						
**White**	702 (93%)	335 (95%)	—	—		
**Black**	32 (4%)	14 (4%)	1.02	0.56,1.87		
**Other**	23 (3%)	3 (<1%)	0.32	0.08,1.30		
**Depression (n)**	138 (18%)	69 (19%)	**1.49**	**1.11,1.99**	**1.54**	**1.14,2.08**
***Liver Disease Factors***						
**MELD (mean)**	21±9	22±9	**1.03**	**1.01,1.04**	1.01	1.00,1.03
**Etiology (n)**						
**Hepatitis C**	255 (34%)	136 (38%)	—	—	—	—
**Alcohol**	142 (19%)	70 (20%)	0.94	0.68,1.31	0.80	0.57,1.11
**NASH**	105 (14%)	55 (15%)	1.03	0.69,1.52	0.82	0.54,1.23
**AIH/PBC/PSC**	132 (17%)	45 (13%)	0.76	0.51,1.11	0.73	0.49,1.09
**Other**	123 (16%)	48 (13%)	0.51	0.32,0.82	**0.45**	**0.28,0.73**
**Hepatocellular carcinoma (n)**	154 (20%)	61 (17%)	1.09	0.79,1.50		
***Transplant Factors***						
**Donor Age (mean)**	45±18	50±19	**1.01**	**1.004,1.02**	**1.01**	**1.0001,1.02**
**DCD donor (n)**	70 (9%)	44 (12%)	1.24	0.86,1.81		
**Living Donor (n)**	147 (19%)	40 (11%)	**0.27**	**0.16,0.48**	**0.42**	**0.23,0.76**
**Non-tacrolimus immunosuppression (n)**	82 (11%)	46 (13%)	**1.55**	**1.10,2.18**		
**Facility after transplant (n)**	170 (22%)	125 (35%)	**2.60**	**1.68,2.86**	**2.15**	**1.65,2.81**

Not significant in univariate or multivariate models: gender, comorbidity index, warm and cold ischemia times bold = statistically significant in regression model. Abbreviations: NASH = non-alcoholic steatohepatitis, AIH = autoimmune hepatitis, PBC = primary biliary cirrhosis, PSC = primary sclerosing cholangitis

**Table 4:** Causes of Death by Depression Status (n = 1115).

Cause of Death	Non-Depressed	Total Depressed
	(N = 908)	(N = 207)
**Total Deaths**	261 (29%)	69 (33%)
**Cardio-pulmonary**	72 (8%, 28%)	17 (8%, 25%)
**Infection/Sepsis**	48 (5%, 18%)	10 (5%, 14%)
**Hemorrhage**	10 (1%, 4%)	1 (<1%, 1%)
**Malignancy**	32 (4%, 12%)	9 (4%, 13%)
**Hepatic Failure**	17 (2%, 7%)	5 (2%, 7%)
**Multi-system Organ Failure**	34 (4%, 13%)	5 (2%, 7%)
**Renal**	11 (1%, 4%)	2 (1%, 3%)
**Noncompliance**	2 (<1%, 1%)	1 (<1%, 1%)
**Withdrawal of Care**	**5 (1%, 2%)**	8 (4%, 12%)*
**Other**	7 (1%, 3%)	3 (1%, 4%)

Shown in columns are N(total column %, % of deaths in the column)

*significantly different using fisher’s exact test

## Discussion

Two themes emerge from this retrospective analysis of a large group of liver transplant recipients. First, these data support accumulating evidence that depression is associated with decreased survival after liver transplant. Secondly, these data add to the literature by investigating two more immediate post-transplant outcomes—discharge disposition and length of stay, both which were significantly associated with pre-transplant depression.

These data are consistent with the literature regarding depression in general populations. Depression has been consistently associated with increased cardiovascular and oncologic risk [13, 14], healthcare utilization [15, 16] and mortality [17, 18]. Several phenomena may contribute to the observed relationship between depression and poor health outcomes. Inflammation and activation of the hypothalamic-pituitary axis (HPA) related to depression can lead to a cascade of ill-effects [19]. Independent of this HPA activation, depression has been associated with medical non-adherence and poor health behaviors [14] which may further explain the connection between depression and poor health outcomes.

Our findings add to existing literature regarding depression in liver transplant recipients. In our previous work, we found that depressive symptoms in the first post-transplant year were associated with decreased long-term survival [10]. The present study, with a larger and more diverse sample, supports the association between depression and decreased long-term survival after transplantation. Additionally, the present study included a large cohort of patients with *pre-transplant* histories, allowing us to predict poor outcomes before the first post-transplant year, potentially allowing us to intervene early in the transplant process to target this high-risk group. Studies of post-transplant depression are inherently unable to assess early transplant outcomes. Thus, the present study adds to existing literature by not only assessing mortality but also intermediate outcomes of disposition and length of transplant stay, outcomes which cannot be evaluated in studies of post-transplant depression.

It is critical to recognize depression as a risk factor for adverse post-transplant outcomes because it is a highly treatable condition. While this data set was retrospective and did not allow for an exploration of treatment response and transplant outcomes, our prior data suggest that pre-transplant pharmacotherapy for depression is associated with decreased acute rejection [11]. Future work is necessary to understand the role of various modalities proven to be effective in managing depression, including both pharmacotherapy and psychotherapy, in improving transplant outcomes., The role of involving of caregivers in mental health care in the transplant setting has yet to be explored[20, 21].Thus, this study suggests a potential role for early psychiatric intervention in improving both early and late transplant outcomes, which should be assessed in a future prospective analysis.

It is also notable that withdrawal of care was found to be a more common cause of death in patients with a history of any depression, regardless of treatment status at the time of transplant. Withdrawal of care could include stopping medications, stopping dialysis, or initiation of palliative care. While the numbers are small and the data retrospective, this finding gives some insight into the mechanism of death for patients with depression. The association between depression and withdrawal of care has been reported in other chronic medical conditions [22].

As with any retrospective, observational study, this study has certain limitations. While the retrospective dataset allowed us to examine a very large sample of all of our centers liver transplant patients over a ten year period, we did not have actual measures of psychiatric symptoms or response to antidepressant treatments. Accordingly, we did not know whether patients were treated by other therapeutic modalities such as psychotherapy. While ICD-9 codes are not the ideal way to capture active depressive symptoms, they have been used frequently in studies of depression in which it is not feasible to obtain symptom measures due to the retrospective nature of the study or the large population [23–26]. ICD-9 codes for depression have been found to have suboptimal sensitivity but high positive predictive values and specificity, suggesting that the control group may have some recipients with undetected depression. This would bias the results away from finding the effects that we found in the present study [27]. It was not possible to use psychosocial evaluations to assess for active depressive symptoms because of the non-uniform timing of the psychiatric evaluation in relation to the transplant date. Additionally, the evaluations were often not always available in the medical record. The retrospective nature of the study limited the granularity of the data regarding pre-transplant depression including full data about the timing of the diagnosis and measures of symptom activity. This close assessment of symptoms to transplantation has not been accomplished in other studies of depression pre-transplantation partly due to the unpredictable timing of organ availability in relationship to clinic visits. An additional limitation was our inability to measure and assess cognitive impairment due to the retrospective nature of the study. Delirium and cognitive impairment have been associated with hospital discharge to facilities in other contexts and would be valuable to collect in future prospective assessments of factors associated with discharge disposition [28]. While there were limitations of this dataset in terms of exposure, the transplant outcomes data were captured systematically and meticulously for regulatory reporting and for quality assurance purposes. Another potential limitation of this cohort is that it is comprised of predominantly White recipients at a single-center. Thus, these findings will require validation in a multicenter population using prospective design with more detailed assessment of psychiatric symptoms and symptom severity in a more heterogeneous population. Despite these limitations, these data, together with other studies, suggest that depression is a risk factor for adverse transplant outcomes and add to the literature by adding early post-transplant outcomes of discharge disposition and length of stay to traditional survival assessments.

This study demonstrates that a history of depression is an important marker for worse outcomes after liver transplantation. Future studies are needed to assess the impact of psychotherapeutic and other interventions on outcomes.
